# Sonography of the Primary Cutaneous Melanoma: A Review

**DOI:** 10.1155/2012/814396

**Published:** 2012-03-01

**Authors:** Ximena Wortsman

**Affiliations:** Department of Radiology, Clinica Servet, Faculty of Medicine, University of Chile, Almirante Pastene 150, Providencia, Santiago, Chile

## Abstract

The diagnosis and management of primary cutaneous melanoma have traditionally relied on clinical and histological characteristics. Nevertheless, in recent years there has been a significant growth in the usage of ultrasound for studying the cutaneous layers. Thus, the present paper focuses on the primary lesion, its sonographic characteristics, the potential benefits of early imaging, and the new developments on the ultrasound field applied to cutaneous melanoma.

## 1. Introduction

Cutaneous malignant melanoma constitutes 4 to 11% of all skin cancers but is responsible for more than 75% of skin cancer-related deaths producing more than 8000 deaths per year in the United States [1]. To date the diagnosis is clinical, and the usage of ultrasound in the study of cutaneous melanoma has been focused more on the locoregional staging than on the primary lesion, and the prognosis being mainly assessed by clinical and histological features.

The staging of melanoma is mostly based on the Breslow classification that relies on sequential tumor infiltration (depth) by histology within the different cutaneous layers, providing a measurement of the microscopic invasion from the stratum granulosum of the epidermis to the deepest portion of the tumor [2]. The Breslow index has been reported to correlate well with the prognosis of the disease. Moreover, according to the thickness of the primary tumor, important decisions are taken such as the size of the excision and the free margins or the requirement for a sentinel lymph node procedure.

Nevertheless, there are controversial reports about the appropriate size of the excision that should be performed on melanoma. Hence, a small (but potentially important) difference in overall survival between wide and narrow excision margins has been reported that cannot be confidently ruled out. Literature has mentioned that recurrence-free survival is favored with wide excision (Hazard Ratio 1.13; *P* = 0.06; 95% confidence interval 0.99 to 1.28) but the results have not reached statistical significance (*P* < 0.05 level). Furthermore, randomized trial evidence seems to be insufficient to address optimal excision margins for primary cutaneous melanoma [3].

Reports on recurrence rates in melanoma have shown a wide range depending on the stage of the primary tumor; thus, they can vary from 7.1% in stage I to 51% in stage III [4]. Moreover, older patients with thicker tumors and angiolymphatic invasion appear to be at higher risk for local and in-transit recurrence [5]. Hence, the appearance of in-transit metastasis seems to be linked to the biological characteristics of the tumor cells rather than an influence of the surgical technique [6].

Recent advances in ultrasound technology have allowed observation of the cutaneous layers with good resolution. Thus, using multichanneled color Doppler machines, with variable frequency probes that reach frequencies ≥15 MHz, the echostructure of the skin layers can be clearly defined [7]. Furthermore, in spite of the limitations of sonography to detect pigments such as melanin, the noninvasive assessment of the characteristics of the primary tumor, such as thickness and blood flow, may contribute to the modification of critical management decisions.

The literature has sequentially increased sonographic information about primary cutaneous melanoma lesions [7–9]. The first sonographic reports on melanoma were performed using other type of ultrasound equipment that use fixed high-frequency probes that go from 20 to 100 MHz. The usage of these machines provided valuable information that showed a good correlation between sonometry and histometry (*r* = 0.88), with a mean difference of 0.39 mm (relative difference 28%). This report has also shown that tumors measuring between 0.55 and 0.95 mm thick were incorrectly classified according to histology in 34%. Nevertheless, thicker tumors that measured between 1.30 and 1.70 mm were incorrectly classified in 50% of cases [10]. The latter results may perhaps be related to the low penetration of these fixed frequency ultrasound machines (approximately 6 mm at 20 MHz, 3 mm at 75 MHz, and 1 mm at 100 MZ) [11, 12]. This point could be relevant since the skin presents variable thickness of its layers according to the corporal region; thus, only the dermis could measure more than 3.0 mm (thickness) in normal individuals such as, for example, in the dorsal thoracic region [7]. Nevertheless, the usage of 75 MHz fixed high-frequency ultrasound for observing thin melanomas that presented a mean histological Breslow thickness of 0.4 mm (22 were in situ) showed a high correlation (Pearson's *r* = 0.908, *P* < 0.001) with a median percentage error up to 13% with the histological Breslow thickness. Sonographic measurements in this report were considered as highly reliable for invasive melanoma, even in the presence of lymphocytic infiltration or nevus [12].

On the other hand, using multichanneled color Doppler ultrasound equipment with variable frequency probes that go from 10 to 15 MHz, it has already been reported that ultrasound is capable of differentiating melanomas measuring < or >1 mm of thickness, which is important for requiring, for example, a sentinel lymph node procedure that is indicated in melanomas measuring more than 1 mm thick [13, 14].

Thus, recognition of this vital depth information can support the performance of “one-time” surgery in melanoma, where the size of the incision and the free margins as well as the sentinel lymph node procedure are planned with an imaging support presurgically [15]. The use of sonography as a “thickness discriminator” has shown high correlation with histological results [16–18]. Nevertheless, sonographic measurements can be slightly higher than histology because they correspond to *in vivo* tissue without dehydration or fixation, and may include subtumoral mononuclear infiltrate and nest of nevus cells which cannot be differentiated sonographically from melanoma cells [10]. This small difference in the measurements has been reported to be much less than the actual size of the extension used for assessing the free margins that is usually used in melanoma surgery. Moreover, using 12–15 MHz frequency, the sensitivity and positive predictive values of ultrasound in detecting lesions thicker than 1 mm has been reported to be 92% and 95%, respectively [18].

Although early stages can be successfully treated by surgery, adjuvant therapies are used for managing advanced states. Furthermore, exceptions to wide local excision include cases where surgical excision may be cosmetically disfiguring or associated with increased morbidity and mortality. Thus, there are several meta-analyses of randomized controlled trials that have reported the impact of adjuvant interferon alfa therapy for malignant melanoma [19–23]. Also, there are systemic therapies such as cytokine therapy, chemotherapy, and biochemotherapy. Additionally, cellular therapy, gene therapy and targeted therapy are undergoing investigation [24]. Furthermore, the role of definitive or adjuvant radiotherapy has largely been relegated to palliative measures. However, the emerging clinical and radiobiological data suggest that many types of effective radiation therapy, such as intensity-modulated radiotherapy for melanoma of the head and neck, and adjuvant radiotherapy for selected high-risk, node-positive patients can improve outcomes [25]. Hence, these nonsurgical treatments may also require noninvasive imaging modalities for objective monitoring of the anatomical changes invisible to the naked eye. Moreover, new applications of ultrasound such as sonodynamic therapy (SDT), that use the energy of ultrasound and certain chemical photocatalyst sensitizers such as Titanium dioxide (TiO_2_), can be used to kill the cancerous cells in melanoma. So far, this sonication procedure is performed by experimental devices but in the future, perhaps, may be adapted to our usual machines [26].

## 2. Sonographic Characteristics of the Primary Melanoma

**Figure 1:**
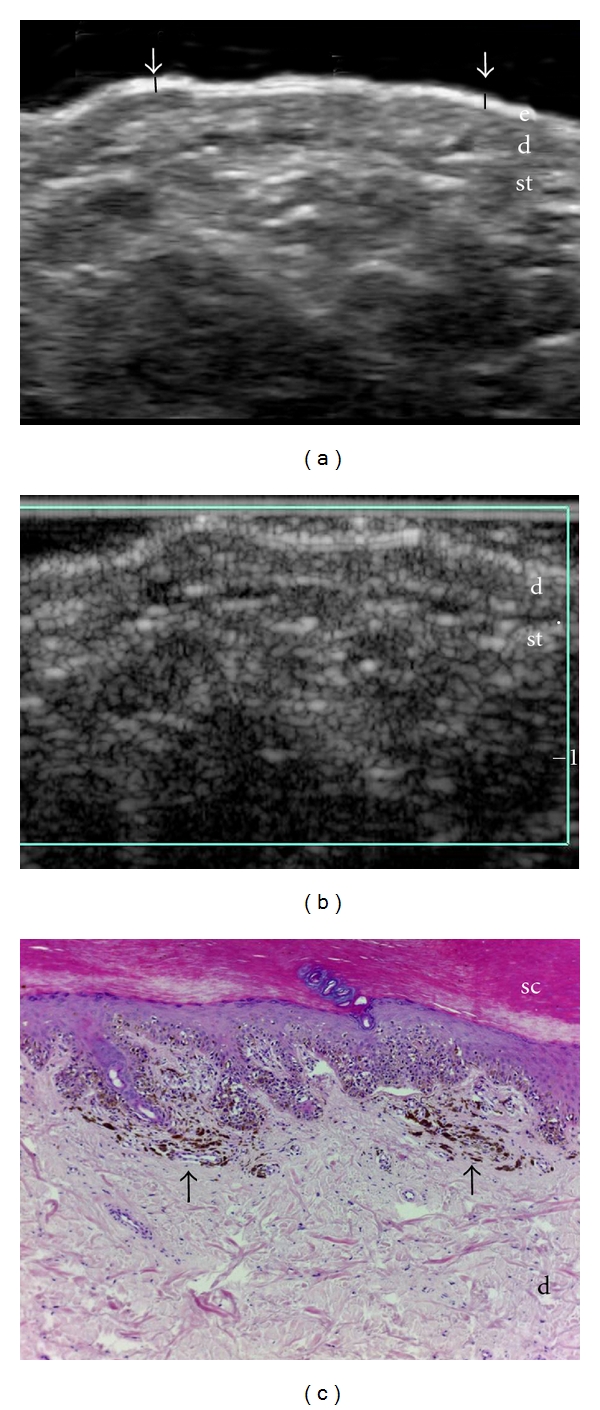
Melanoma in situ. (a) Ultrasound (transverse view, plantar region) shows lack of detectable solid tumor. Notice the partial loss of the normal bilaminar hyperechoic pattern of the plantar epidermis in parts of the lesion (arrows). (b) Color Doppler ultrasound demonstrates lack of hypervascularity at the lesional site. (c) Histology (HE × 100, acral skin, courtesy of Dr. Laura Carreño, Department of Pathology, Hospital Clinico Universidad Chile, University of Chile, Santiago, Chile) shows atypical nests of melanocytic cells within the epidermis (in situ). At the dermis there are some melanophages (arrows). Abbreviations: sc: stratum corneum; d: dermis; st: subcutaneous tissue.

**Figure 2:**
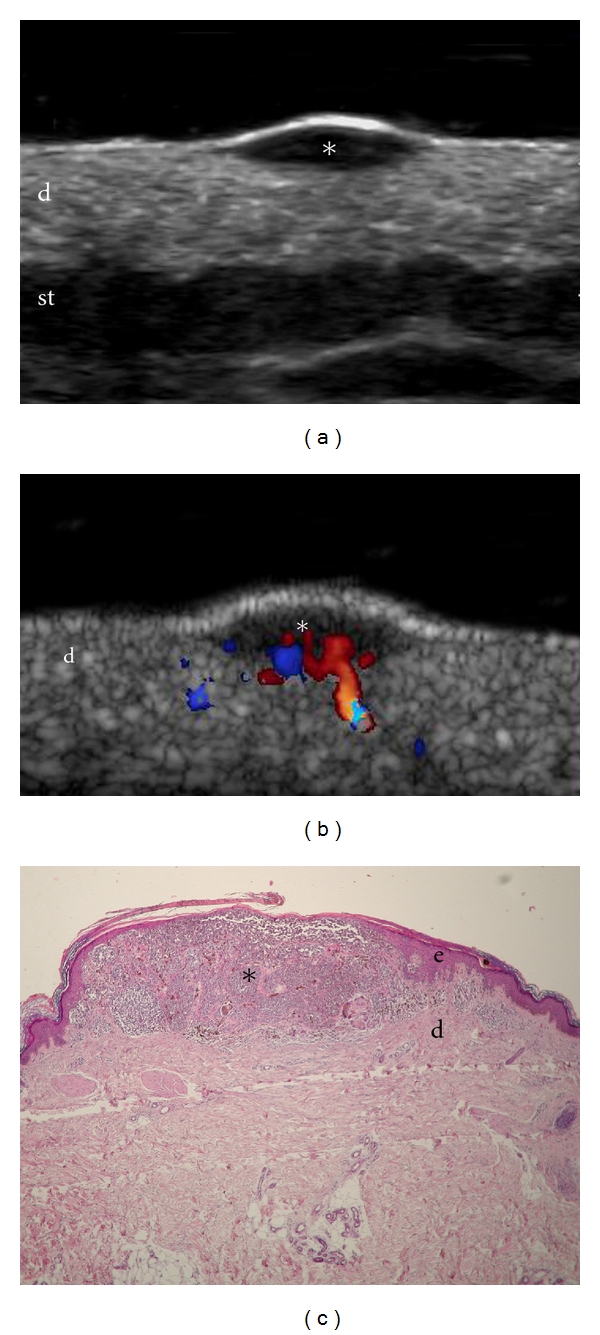
Melanoma primary tumor infiltrating dermis. (a) Ultrasound (grey scale, transverse view, dorsal region) shows oval-shaped hypoechoic lesion (∗) that involves the dermis. The epidermis also appears more hyperechoic and thickened. (b) Color Doppler ultrasound (transverse view) demonstrates increased blood flow within the tumor that predominates at the bottom of the lesion (∗). (c) Histology (HE × 4, courtesy of Dr. Laura Carreño, Department of Pathology, Hospital Clinico Universidad Chile, University of Chile, Santiago, Chile) demonstrates atypical melanocytic cells infiltrating epidermis and dermis. Prominent vessels are detected in the subtumoral area. Abbreviations: e: epidermis; d: dermis; st: subcutaneous tissue; ∗: melanoma site.

**Figure 3:**
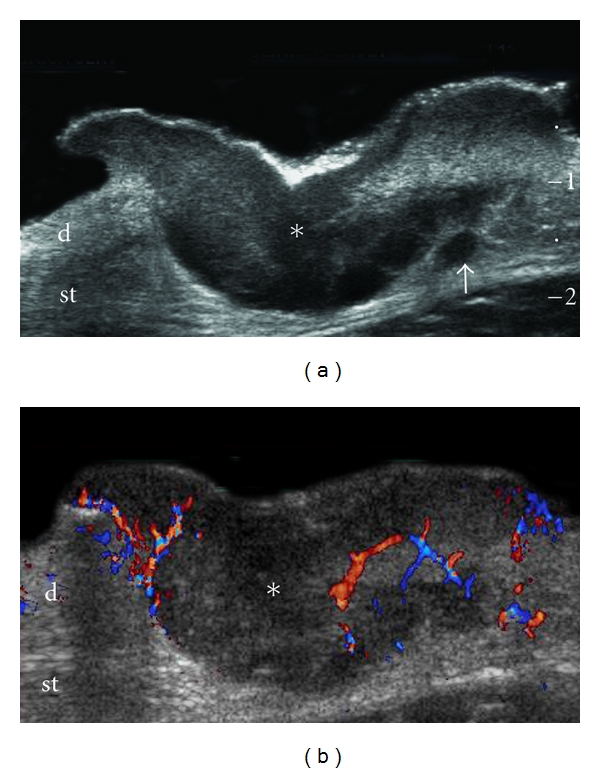
Bulky melanoma at the abdominal wall. (a) Ultrasound (transverse view) shows fusiform hypoechoic lesion (∗) infiltrating dermis and subcutaneous tissue. Notice the hypoechoic solid nodule besides the primary tumor that corresponds to a satellite metastasis (arrow). (b) Color Doppler ultrasound demonstrates increased blood flow within the lesion. Abbreviations: d: dermis; st: subcutaneous tissue.

**Figure 4:**
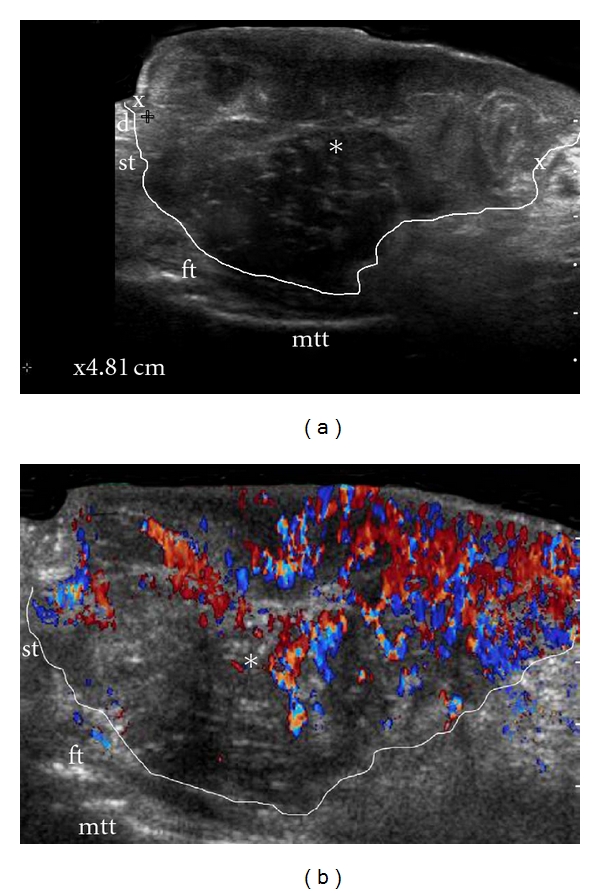
Bulky melanoma at the plantar region. (a) Ultrasound (longitudinal view) shows heterogeneous mass (∗, and outlined) infiltrating dermis and subcutaneous tissue, almost reaching the flexor tendon (fl) close to the second metatarsal bone (mtt). (b) Color Doppler ultrasound shows highly increased blood flow covering most parts of the lesion.

On sonography, early lesions usually present oval or fusiform shape and hypoechogenicity [27, 28]. Commonly, these lesions infiltrate the dermis and show increased blood flow within the tumor. Nevertheless, cases with in situ lesions may not show detectable masses. In cases with ulcerations, the epidermis may be irregular or discontinuous, and increased echogenicity of the surrounding subcutaneous tissue may also be found [9]. Since the tumor can show asymmetry in its shape, the measurement of thickness should be performed at the deepest point. When examining these lesions, it is recommended to apply a copious amount of gel on the skin surface; thus we can observe the lesion without compressing the vascularity that may present prominent low velocity arterial vessels within the lesions, especially in bulky tumors. Knowledge of the peak systolic arterial velocity of these vessels (cm/sec) may provide an idea of the angiogenic power of the tumor that can correlate with its metastatic potential. Additionally, preoperative ultrasound and guided fine needle aspiration cytology have been reported as useful for identifying sentinel nodes metastasis and reduce the need for surgical sentinel nodes procedures. Moreover, peripheral perfusion has been mentioned as an early sign of involvement and of crucial importance to achieve a high identification rate of metastasis. Balloon shape and loss of central echoes are indeed late signs of malignant nodal infiltration [29, 30]. When studying bulky melanomas, the use of lower frequency probes can help to obtain the complete extension of the tumor which may also require extended field of view capabilities. Of the settings, the power Doppler that is usually more sensitive for detecting slow flow could be more useful than the color Doppler mode. The lowest pulse repetition frequencies (that do not cause aliasing or excessive reduction of the frame rate) and the lowest wall filter and color gain, below the noise threshold, are preferred to obtain better quality images [9, 31] (Figures 1, 2, 3, and 4).

Commonly close to the primary lesion, satellite lesions (arising within 2 cm from the primary tumor) may be found, which may appear as hypoechoic solid masses in the subcutaneous tissue, presenting variable degrees of vascularity [9, 31] (Figure 3).

## 3. Contrast-Enhanced Ultrasound on Melanoma

Contrast enhanced ultrasound has been progressively more used in the study of the primary lesion and the locoregional metastases. The usage of contrast may support the nonsurgical treatment of the primary lesion and clarify a doubtful cortical thickening at the lymph nodes [32, 33]. Moreover, vascular density has already been correlated with metastatic potential using color Doppler ultrasound. Thus, assessment of the tumor perfusion characteristics is becoming an issue in oncology. Furthermore, neovascularization is a prognostic factor for metastasis equivalent to the Breslow index [33]. Therefore, an early provision of functional images of the primary tumor can provide relevant information concerning the usefulness of new targeted therapies and antiangiogenic treatments, thereby allowing an early assessment of the effectiveness of the treatment [34, 35]. This functional analysis of the tumoral perfusion relies on the acoustic properties of the microbubbles that increase the signal-to-noise ratio and the sensitivity to microvessel detection at higher levels than the power Doppler [36].

## 4. Sonoelastography in Cutaneous Melanoma

As yet, few reports that show the usefulness of sonoelastography in cutaneous melanoma are available. To date, the reports are mostly based on acoustooptical elastography (AOE), an experimental imaging modality for quantifying the mechanical behavior of skin lesions. The method relies upon stimulating the tissue with a low-frequency acoustic force and imaging the resulting strains in the tissue by means of quantifying the magnitude of the dynamic shift in a backreflected laser speckle pattern from the skin. The magnitude of the shift reflects the local stiffness of the tissue, which has shown promising results when comparing benign melanocytic nevi with melanoma [37].

## 5. Conclusion

Ultrasound can be a reliable tool for assessing relevant characteristics of the primary lesion such as depth and vascularity. This imaging method is not intended to replace histology but perhaps the anatomical information can provide a missing link between the clinical evaluation, and the later, biopsy and treatments. Furthermore, this detailed data has the potential to help with deciding on appropriate treatment and therefore, may allow a one-time treatment, support an early test of the efficacy of a treatment, and perhaps help to decrease the recurrence rates. Nowadays, considering the fast evolution of imaging technologies, the inclusion of imaging modalities in the routine evaluation and classification of the primary tumors could be the next step to follow in the management of melanomas.
